# Complex Medium-Chain Triglycerides Mitigate Porcine Epidemic Diarrhea Virus Infection in Piglets by Enhancing Anti-Inflammation, Antioxidation, and Intestinal Barrier Function

**DOI:** 10.3390/v17070920

**Published:** 2025-06-27

**Authors:** Tingting Hu, Yunhao Liu, Sihui Gao, Xiaonan Zhao, Huangzuo Cheng, Youjun Hu, Huaqiao Tang, Zhiwen Xu, Chunlin Fang

**Affiliations:** 1 Department of Animal Husbandry and Veterinary Science, Chengdu Agricultural College, Chengdu 611130, China; hutt0409@163.com; 2 College of Veterinary Medicine, Sichuan Agricultural University, Chengdu 611130, China; 13281342999@163.com (Y.L.); 17313152250@163.com (S.G.); turtletang@163.com (H.T.); abtcxzw@126.com (Z.X.); 3 Innovation Center of Guangdong Nuacid Biotechnology Co., Ltd., Qingyuan 511545, China; zxn780213675@126.com (X.Z.); chz868@88.com (H.C.); 61368851@163.com (Y.H.)

**Keywords:** porcine epidemic diarrhea virus, complex medium-chain triglycerides, glyceryl tricaprylate/caprate, glycerol monolaurate, piglets, intestinal barrier functions

## Abstract

Porcine epidemic diarrhea (PED), a highly contagious enteric disease caused by the porcine epidemic diarrhea virus (PEDV), is characterized by vomiting, diarrhea, and dehydration, leading to high mortality in newborn piglets and significant economic losses in the swine industry. The shortage of effective PED vaccines emphasizes the need to explore potent natural compounds for therapeutic intervention. It has been shown that glycerol monolaurate (GML) effectively inhibits PEDV replication in vivo and in vitro. Further investigation is needed to assess whether complex medium-chain triglycerides (CMCTs), composed of glyceryl tricaprylate/caprate (GTCC) and GML, offer an efficient anti-PEDV activity. In this study, piglets were orally infected with PEDV and exhibited typical clinical signs, including diarrhea and vomiting, accompanied by intestinal inflammation, oxidative stress, and tissue damage. CMCTs were administered orally twice daily for one week. In vivo findings indicate that CMCT treatment alleviated clinical signs and prevented weight loss. It significantly increased serum immunoglobulins (IgG, IgM, and IgA) and intestinal mucosal sIgA and MUC-2 levels, while reducing pro-inflammatory cytokines (IL-1β, IL-6, TNF-α, and IL-17) and increasing antiviral interferons (IFN-α and IFN-γ), anti-inflammatory cytokines (IL-4 and IL-10), and IL-22. Antioxidant enzyme activities (T-AOC, SOD, GSH-Px, and CAT) were elevated, whereas oxidative stress markers (iNOS, NO, and MDA) were decreased. Expression of intestinal tight junction proteins claudin-1 and ZO-1 was restored. Moreover, CD4^+^ and CD8^+^ T cell populations increased, and the functions of regulatory T cells (Tregs) were restored. Gut microbiota analysis showed increased beneficial genera (*Streptococcus* and *Ligilactobacillus*) and decreased pathogenic *Escherichia-Shigella*. These results demonstrate that CMCTs mitigate PEDV infection by enhancing anti-inflammation, antioxidation, and intestinal barrier function, as well as modulating gut microbiota composition. This study improves the understanding of the pathogenesis of PEDV and highlights CMCTs as a promising therapeutic candidate for PED.

## 1. Introduction

Porcine epidemic diarrhea (PED) is an acute, highly contagious enteric disease in swine caused by the porcine epidemic diarrhea virus (PEDV), a member of the genus *Coronavirus* within the family *Coronaviridae*. PEDV primarily infects the digestive tract of pigs, causing vomiting and diarrhea, and leading to particularly high mortality in piglets [1]. As the largest digestive and absorptive organ, the intestine is the body’s primary line of resistance to bacterial and viral invasion in pigs. The intestinal barrier functions in pigs are a complex system comprising four barriers: mechanical, biological, immune, and chemical [2,3]. Extensive viral replication in the intestinal epithelium results in significant damage, including marked villous atrophy, loss of enterocytes and goblet cells, destroyed tight junctions, and reduced mucin production. This damage facilitates the breach of the intestinal barrier by bacteria and toxins, which in turn induces the production of various inflammatory cytokines and leads to intestinal inflammation and oxidative stress [4,5]. Hence, it is crucial to preserve the intestinal barrier’s functions to resist PEDV infection.

Glyceryl tricaprylate/caprate (GTCC) is a triacylglycerol composed of caprylic (C_8_) and capric (C_10_) acids, two medium-chain fatty acids. As a specialized MCT, GTCC exhibits high stability, efficient absorption, and rapid energy metabolism, thereby promoting cellular repair and functional maintenance [6,7]. Glyceryl monolaurate (GML) is a monoglyceride derived from lauric acid, a medium-chain fatty acid (C_12_) commonly found in coconut oil and palm kernel oil. It possesses antimicrobial [8] and antiviral properties, significantly inhibiting enveloped viruses, including human immunodeficiency virus type 1 (HIV-1) [9] and African swine fever virus (ASFV) [10,11]. Beyond its antiviral effects, GML can modulate intestinal oxidative stress, counteract intestinal inflammation, and regulate the intestinal microbiota to maintain intestinal health, thereby reducing pathogenic microorganism infections in the intestinal tract [12,13,14,15].

Currently, a primary approach to controlling PEDV infection is vaccination. However, the immature immune systems of piglets may limit the efficacy of PED vaccines [16]. Consequently, this situation poses significant challenges for managing PED in piglets due to the lack of effective treatments. Therefore, this study aims to evaluate the protective potential of complex medium-chain triglycerides (CMCTs) against PEDV infection in piglets and elucidate their roles in regulating the intestinal microenvironment to enhance intestinal barrier functions.

## 2. Materials and Methods

### 2.1. Virus and Animals

The PEDV SC strain was supplied by the Animal Biotechnology Center of Sichuan Agricultural University. Twenty-five experimental piglets (Landrace × Yorkshire, one week old), all healthy and of similar body weight, were obtained from Chengdu Wangjiang Agriculture and Animal Husbandry Technology Co., Ltd. (Chengdu, China; a PEDV-free farm). These piglets were not vaccinated with a PED vaccine. The experimental milk replacer, used as the basal diet, was purchased from Tongwei Group Co., Ltd. (Chengdu, China) and confirmed to be free of anti-PEDV antibodies.

### 2.2. Drugs and Reagents

The CMCTs (consisting of GTCC and GML at a ratio of 2:1) were prepared by Nuacid Nutrition Co., Ltd. (Qingyuan, China). ELISA kits for IgG, IgM, IgA, and MUC-2, secretory IgA (sIgA), and IL-6, IL-1β, TNF-α, IL-4, IL-10, IFN-α, IFN-γ, IL-17, and IL-22 were purchased from Jiangsu Meimian Industrial Co., Ltd. (Yancheng, China). RIPA lysis buffer was obtained from Beyotime Biotechnology (Shanghai, China). The BCA Protein Assay Kit was purchased from Nanjing Vazyme Biotech Co., Ltd. (Nanjing, China). T-AOC, CAT, SOD, iNOS, GSH-Px, and MDA were supplied by Nanjing Jiancheng Bioengineering Institute (Nanjing, China). RNAiso Easy, PrimeScript™ RT reagent Kit (Perfect Real Time) and TB Green^®^ Premix Ex Taq™ (Tli RNaseH Plus, Kusatsu, Japan) were supplied by Takara Biomedical Technology Co., Ltd. (Beijing, China). Immunofluorescence antibody was purchased from ABclonal Technology (Wuhan, China). All other chemicals and reagents were analytically pure and purchased from standard commercial suppliers.

### 2.3. Piglet Infection Model and Treatments

Prior to the experiment, rigorous disinfection measures were implemented at the experimental site, including overnight formaldehyde fumigation and subsequent spraying with a broad-spectrum disinfectant. After acquiring the piglets, continuous attention was given to maintaining their hygiene, and they were fed a milk replacer optimized for growth. A 3-day observation period was conducted to ensure the health of the piglets before starting the experiment. Twenty-five one-week-old piglets were randomly assigned to five groups: Control (unchallenged, untreated), Model (PEDV-challenged, untreated), CMCTs high/medium/low (PEDV-challenged, treated with high, medium, or low doses of CMCTs, respectively). Piglets in the Model and CMCTs groups were orally inoculated with 5 mL of PEDV stock at 10^5^ TCID_50_/mL per piglet, while those in the Control group received an equivalent volume of sterile saline. The PEDV dose (10^5^ TCID_50_/mL, 5 mL) was chosen based on previous studies to induce infection [17]. Immediately after the PEDV challenge, piglets in the high (2.0 g/kg), medium (1.0 g/kg), and low (0.5 g/kg) dose CMCT groups received oral administration of the treatments twice daily for 7 consecutive days. The CMCTs were suspended in sterile normal saline and thoroughly mixed to ensure uniform dispersion and accurate dosing. The piglets in the model group received an equal volume of sterile normal saline following the same schedule.

### 2.4. Clinical Observation and Sample Collection

The control and infected groups were housed in separate rooms under strict biosecurity protocols. A unidirectional workflow was implemented, beginning with the control room and proceeding to the infected room to minimize the risk of cross-contamination. Protective clothing and footwear were changed between rooms, and hands and equipment were disinfected prior to each entry. Clinical signs, including diarrhea, dehydration, and vomiting, were monitored daily, and body weights were recorded every other day throughout the 7-day observation period. At 7 days post-infection and treatment, all piglets were euthanized for sample collection. For animals that developed severe clinical signs prior to the scheduled endpoint, tissue and serum samples were collected immediately upon reaching a moribund state to prevent sample loss and maintain a consistent sample size across groups. Euthanasia was performed under inhalation anesthesia using isoflurane, with the concentration gradually increased to 4–5% in 100% oxygen until deep anesthesia was achieved. Exsanguination via the anterior vena cava was then carried out to complete euthanasia. Blood, intestinal mucosa, tissues, and intestinal contents were collected; some samples were fixed in 4% paraformaldehyde, while others were snap-frozen and stored at −80 °C for subsequent analyses.

### 2.5. Histological Analysis

The intestinal tissues were fixed with 4% paraformaldehyde. Subsequently, the fixed tissues were sectioned, dehydrated, embedded in paraffin, and stained with hematoxylin and eosin (H&E). A light microscope was utilized to observe histopathological changes and capture images.

### 2.6. Serum Immunoglobulin Quantification

After natural coagulation, blood was centrifuged (1000× *g*, 4 °C, 10 min) in a refrigerated centrifuge to separate the serum. Immunoglobulin (IgG, IgM, and IgA) concentrations in the serum were assessed using commercial ELISA kits.

### 2.7. Quantification of MUC-2 and sIgA in Intestinal Mucosa

Porcine jejunal mucosa was carefully collected, transferred to sterilized centrifuge tubes, and homogenized in PBS (1:5). The homogenate was then centrifuged at 8000× *g* at 4 °C for 15 min to obtain the supernatant, which was used to assess the protein expression of mucins (MUC-2) and secretory IgA (sIgA) in the intestinal mucosa.

### 2.8. Jejunal Cytokine Expression Analysis

PEDV primarily infects the small intestine of pigs, with a particular predilection for the jejunum and ileum regions, resulting in severe atrophic enteritis [18]. To examine the protective effect of CMCTs on the porcine small intestine, we used homogenized jejunal tissues (PBS, 1:5), which were then centrifuged at 8000× *g* at 4 °C for 15 min. The supernatant was utilized to measure cytokine concentrations (IL-6, IL-1β, TNF-α, IL-4, IL-10, IFN-α, IFN-γ, IL-17, and IL-22) in the jejunal tissues by ELISA kits.

### 2.9. Jejunal Oxidative Stress Analysis

Jejunal tissues were homogenized in RIPA lysis buffer to extract total proteins. The homogenate was then centrifuged at 12,000× *g* and 4 °C for 15 min. Protein concentrations were measured using a bicinchoninic acid (BCA) assay kit according to the manufacturer’s instructions. The concentrations of oxidative stress markers, including T-AOC, CAT, SOD, iNOS, GSH-Px, and MDA, were measured using commercial kits. These assessments were conducted to evaluate the effect of CMCTs on oxidative stress in PEDV-infected intestinal tissues.

### 2.10. Indirect Immunofluorescence Analysis

The intestinal tissues were fixed in 4% paraformaldehyde and then embedded in paraffin. Immunofluorescence staining was used to assess the protein expression of claudin-1 and ZO-1, as well as the colonization of T cells and their subsets, identified by T cell surface markers CD127, CD4, and CD8. After sectioning, the tissues underwent deparaffinization, rehydration, and blocking. Primary antibodies targeting the proteins were incubated overnight at 4 °C. Fluorescently labeled secondary antibodies were then applied and incubated. To visualize cell nuclei, DAPI was used as a counterstain. The images were captured using a Leica microscope and analyzed with the ImageJ software (v1.8.0).

### 2.11. 16S rRNA Sequencing of Gut Microbiota

The jejunal contents were collected and transported to Novogene Co., Ltd. (Tokyo, Japan) in dry ice. Total DNA was extracted using a DNA extraction kit following the manufacturer’s protocols. The concentration, purity, and quality of the extracted DNA were then evaluated. The primers 341F (5′-CCTAYGGGRBGCASCAG-3′) and 806R (5′-GGACTACNNGGGTATCTAAT-3′) were utilized to amplify the V3-V4 domain of the microbial 16S rRNA gene via PCR. The amplified products were sequenced using the Illumina MiSeq platform. Data processing was then performed using the R programming language, involving analysis of intergroup microbial abundance, diversity, clustering, and other pertinent information.

### 2.12. Statistical Analysis

All data were presented as means ± SD and analyzed using SPSS v.22.0 (IBM Corp., Armonk, NY, USA). One-way ANOVA was employed to compare groups, followed by Tukey’s post hoc test. For 16S rRNA sequencing, differential microbiota were identified using the Wilcoxon rank-sum test (for two groups) or the Kruskal–Wallis H test with Bonferroni correction (for multiple groups) with a 95% confidence interval. Statistical significance was indicated by * *p* < 0.05, ** *p* < 0.01, *** *p* < 0.001, and **** *p* < 0.0001.

## 3. Results

### 3.1. CMCTs Can Ameliorate Clinical Signs

Piglets infected with PEDV displayed vomiting and watery diarrhea. The model group experienced severe dehydration, emaciation, contamination with vomit and excreta, and death (Table 1). In contrast, the control group showed normal growth. Piglets receiving low-dose treatment exhibited clinical signs similar to the model group but gradually recovered. In the medium-to-high dose treatment group, vomiting was absent, and transient diarrhea was quickly resolved, indicating the effectiveness of the treatment. Additionally, the piglets tolerated CMCTs well, showing no signs of toxicity.

### 3.2. CMCTs Prevent Weight Loss in Infected Piglets

**Figure 1:**
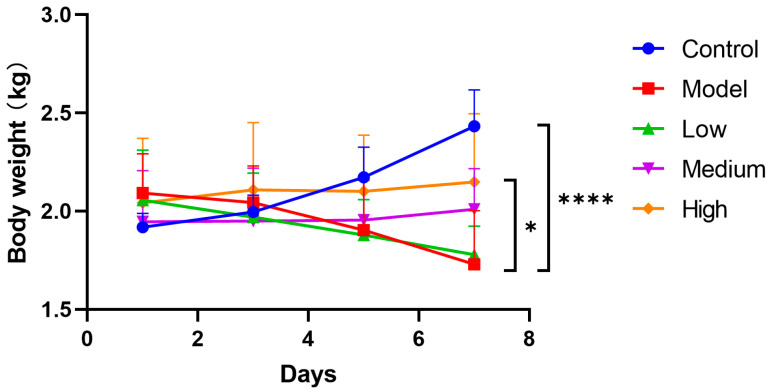
Effects of CMCTs on piglet body weight after PEDV infection. After the challenge, the treatment groups received low (0.5 g/kg), medium (1.0 g/kg), and high (2.0 g/kg) doses of CMCTs orally twice daily for 7 days, with body weight measured every other day. Data were presented as means ± SD (n = 5). Statistical analyses were performed using two-way ANOVA followed by Dunnett’s post hoc test. * *p* < 0.05, ** *p* < 0.01, *** *p* < 0.001, **** *p* < 0.0001, compared with the model group.

After infection, piglets in the model group rapidly developed dehydration, appetite reduction, and persistent weight loss without recovery. In contrast, control group piglets maintained a normal appetite and gained weight. The medium and high dose treatment groups exhibited improved appetite and regained physical strength, initiating weight gain by the fourth day post-treatment (Figure 1). The results indicated that CMCTs effectively slowed weight loss in piglets and promoted the restoration of their growth performance.

### 3.3. CMCTs Effectively Suppressed PEDV-Induced Inflammation and Small Intestine Damage

**Figure 2:**
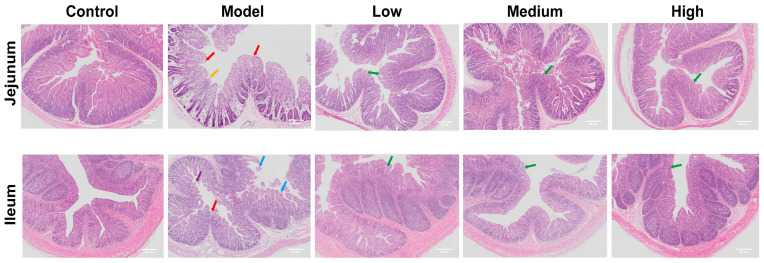
The effects of CMCTs on piglet jejunum and ileum. Histological analysis of H&E staining images. Scale bar, 50 μm. Magnification, 50×. Yellow arrows: mucosal thinning; red arrows: villous atrophy; blue arrows: epithelial exfoliation; purple arrows: necrotic cellular debris; green arrows: restored villous structure.

Compared to the control group, the model group exhibited pronounced histopathological alterations in the jejunum and ileum, including severe mucosal thinning (yellow arrows), villous atrophy (red arrows), and epithelial exfoliation (blue arrows). Necrotic cellular debris was also evident within the intestinal lumen (purple arrows). Post-treatment, these lesions in the jejunum and ileum were markedly ameliorated, with a dose-dependent restoration of villous architecture observed (green arrows) (Figure 2).

### 3.4. CMCTs Enhance Serum Immunoglobulin Levels in Infected Piglets

**Figure 3:**
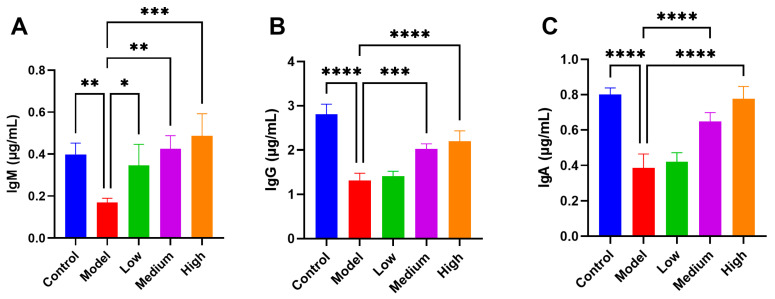
Effects of CMCTs on piglet serum immunoglobulin levels. Concentrations of three blood immunoglobulins, IgM (**A**), IgG (**B**), and IgA (**C**) in the serum. Data were presented as means ± SD (n = 5). Statistical significance was determined using one-way ANOVA, followed by Dunnett’s post hoc test. * *p* < 0.05, ** *p* < 0.01, *** *p* < 0.001, **** *p* < 0.0001, compared with the model group.

The levels of serum immunoglobulins reflect the general humoral immune status of the host. PEDV infection in piglets led to a significant reduction in total IgG, IgM, and IgA concentrations. However, administration of CMCTs significantly increased IgM levels in a dose-dependent manner (Figure 3A), suggesting a potential role for CMCTs in promoting the early activation of humoral immunity. In addition, CMCTs significantly elevated total IgG (Figure 3B) and IgA (Figure 3C) levels at medium and high doses. These findings indicate that CMCTs may help maintain or restore humoral immune balance during PEDV infection.

### 3.5. CMCTs Augment the Expression of Intestinal Mucosal MUCs and sIgA in Infected Piglets

**Figure 4:**
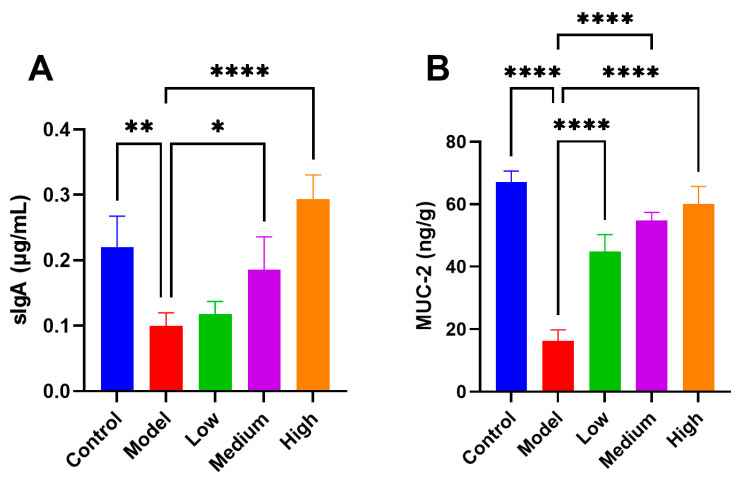
The CMCTs exhibit the capacity to augment the expression of sIgA and MUC-2 in the jejunal mucosa of piglets. Concentrations of sIgA (**A**) and MUC-2 (**B**) in jejunal mucosa; data were presented as means ± SD (n = 5). Statistical significance was determined using one-way ANOVA, followed by Dunnett’s post hoc test. * *p* < 0.05, ** *p* < 0.01, *** *p* < 0.001, **** *p* < 0.0001, compared with the model group.

In the pigs infected with PEDV, the expression of intestinal mucosal secretory immunoglobulin A (sIgA) and mucins (MUC-2) reduced considerably (Figure 4). sIgA is vital for intestinal immune defense and maintaining intestinal health. Studies indicate that sIgA, secreted by intestinal mucosal epithelial cells, efficiently neutralizes viruses at invasion sites [19]. MUC-2 plays a significant role in preserving the integrity of the intestinal mucosa, shielding the intestine from pathogen invasion [20]. After treatment, sIgA (Figure 4A) and MUC-2 (Figure 4B) showed dose-dependent upregulation under CMCTs induction, significantly strengthening the intestinal barrier’s resistance.

### 3.6. CMCTs Modulate Inflammatory Cytokine and Interferon Expression in Jejunal Tissue

**Figure 5:**
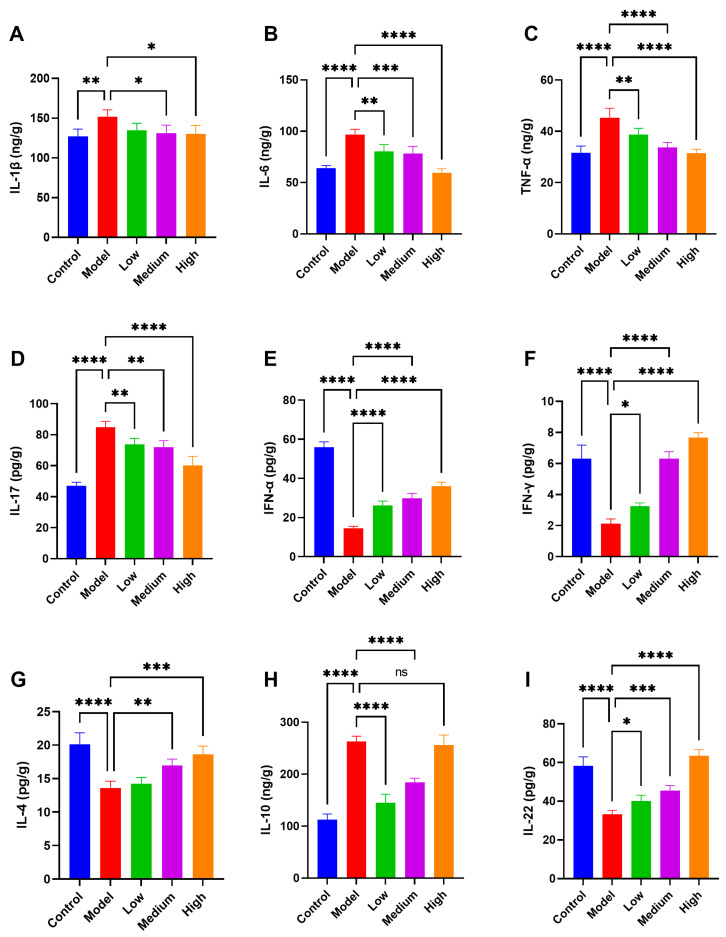
The CMCTs enhance jejunal tissue’s antiviral, anti-inflammatory, and repair factor expression while reducing inflammation. Concentrations of four representative pro-inflammatory cytokines, IL-1β (**A**), IL-6 (**B**), TNF-α (**C**) and IL-17 (**D**); IFN-α (**E**), and IFN-γ (**F**); anti-inflammatory cytokines IL-4 (**G**) and IL-10 (**H**); the intestinal mucosal repair factor IL-22 (**I**) in jejunal tissue. Data were presented as means ± SD (n = 5). Statistical significance was determined using one-way ANOVA, followed by Dunnett’s post hoc test. * *p* < 0.05, ** *p* < 0.01, *** *p* < 0.001, **** *p* < 0.0001, compared with the model group.

After PEDV infection, various pro-inflammatory cytokines, including IL-1β (Figure 5A), IL-6 (Figure 5B), TNF-α (Figure 5C), and IL-17 (Figure 5D), are simultaneously released, resulting in intestinal tissue damage. Treatment with CMCTs in infected piglets significantly decreased the levels of IL-1β, IL-6, TNF-α, and IL-17 in intestinal tissues across all three dosage conditions. These findings indicate that CMCTs effectively mitigate the inflammatory response in infected intestinal tissues, facilitating tissue repair.

PEDV infection significantly decreases the expression of antiviral interferons IFN-α (Figure 5E) and IFN-γ (Figure 5F) in piglet intestinal tissues. CMCTs upregulate these factors in a dose-dependent manner, enhancing piglets’ intestinal resistance to PEDV infection. Moreover, at medium to high doses, the expression levels of anti-inflammatory cytokines IL-4 (Figure 5G), IL-10 (Figure 5H), and the intestinal mucosal repair factor IL-22 (Figure 5I) are significantly increased, bolstering the intestine’s capacity to modulate inflammation and repair tissue damage. CMCTs enhance the expression levels of intestinal antiviral factors, anti-inflammatory factors, and tissue repair factors, thereby strengthening the intestinal defense against infection, reducing inflammation, and promoting tissue repair.

### 3.7. CMCTs Reduced PEDV-Induced Oxidative Stress in Jejunal Tissue

**Figure 6:**
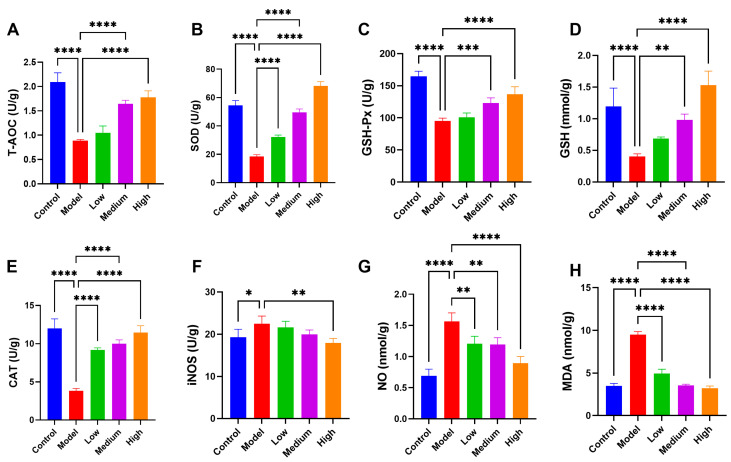
The CMCTs mitigated oxidative stress in the jejunum. Concentrations of T-AOC (**A**), SOD (**B**), GSH-Px (**C**), GSH (**D**), CAT (**E**), iNOS (**F**), NO (**G**), and MDA (**H**) in jejunal tissue from each group. Data were presented as means ± SD (n = 5). Statistical significance was determined using one-way ANOVA, followed by Dunnett’s post hoc test. * *p* < 0.05, ** *p* < 0.01, *** *p* < 0.001, **** *p* < 0.0001, compared with the model group.

Oxidative stress, characterized by an imbalance in oxidative-reduction processes in the body, is a significant contributor to various diseases. Viral infections can induce oxidative stress in cells [21]. The intestine is particularly susceptible to oxidative damage from free radicals [22]. In this research, we found a substantial reduction in antioxidant enzyme levels in the intestines of the PEDV-infected pigs, including total antioxidant capacity (T-AOC), superoxide dismutase (SOD), and glutathione peroxidase (GSH-Px), as well as glutathione (GSH) (Figure 6A–D). Additionally, catalase (CAT) levels were markedly reduced (Figure 6E), while inducible nitric oxide synthase (iNOS), nitric oxide (NO), and the oxidative stress metabolite malondialdehyde (MDA) levels were significantly elevated (Figure 6F–H), indicating pronounced oxidative stress in the intestines following infection. After CMCT therapy, antioxidant enzyme levels significantly increased while oxidative metabolites decreased, indicating enhanced endogenous antioxidant capacity. This therapy notably improved oxidative stress in the intestines of infected pigs, especially at medium to high dosages.

### 3.8. CMCTs Improved the Mechanical Barrier Functions of the Intestine

**Figure 7:**
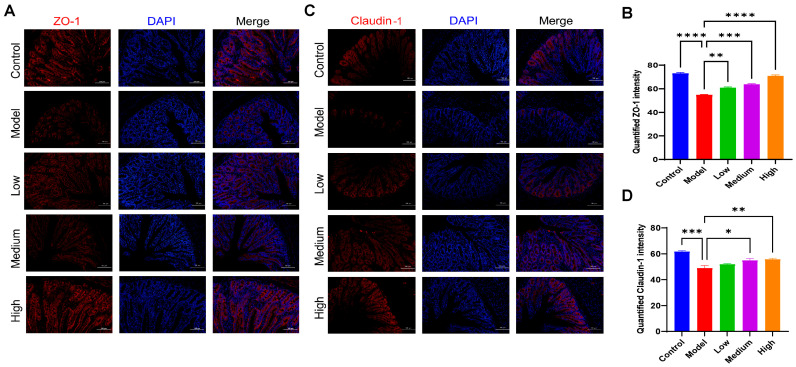
The CMCTs promote the expression of tight junction proteins in the jejunal tissue of piglets. Representative fluorescence microscopy images show ZO-1 (**A**,**B**) and claudin-1 (**C**,**D**) in jejunal tissue. claudin-1 and ZO-1 are shown in red, and nuclei are blue. Scale bar, 100 μm, magnification, 200×. Quantification of fluorescence signals was performed using Image J. Statistical significance was determined using one-way ANOVA, followed by Dunnett’s post hoc test. * *p* < 0.05, ** *p* < 0.01, *** *p* < 0.001, **** *p* < 0.0001, compared with the model group.

Tight junction proteins are fundamental to the mechanical barrier, ensuring the integrity of paracellular permeability and effectively blocking the invasion of pathogens, metabolites, and other toxic substances [23]. The study observed a decrease in the fluorescence expression levels of intestinal tight junction proteins claudin-1 (Figure 7A) and ZO-1 (Figure 7B) in the PEDV-infected pigs. After CMCT administration, the fluorescence expression levels of claudin-1 and ZO-1 significantly increased, indicating that CMCTs confer protective effects on intestinal barrier functions.

### 3.9. CMCTs Improved the Intestinal Immune Barrier Functions

Immune cells are essential components of the intestinal immune defense system [24]. CD4 and CD8 markers identify T helper and cytotoxic T cells, respectively, which are crucial for immune activation and cytotoxicity. CD127 (IL-7 receptor), a marker for regulatory T cells (Tregs), is generally expressed at low levels in Tregs. The elevated expression of CD127 is associated with impaired or suppressed Treg functions [25].

**Figure 8:**
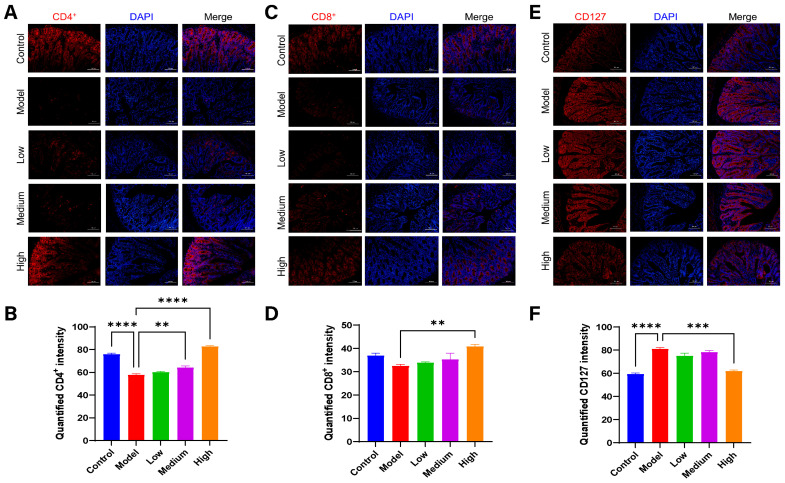
The CMCTs exert a positive regulatory effect on immune cells in the jejunal tissue of piglets. Representative fluorescence microscopy images show CD4^+^ (**A**,**B**), CD8^+^ (**C**,**D**), and CD127 (**E**,**F**) in jejunal tissue. CD4^+^, CD8^+^, and CD127 are shown in red, and nuclei are blue. Scale bar, 100 μm, magnification, 200×. Quantification of fluorescence signals was performed using Image J. Statistical significance was determined using one-way ANOVA, followed by Dunnett’s post hoc test. * *p* < 0.05, ** *p* < 0.01, *** *p* < 0.001, **** *p* < 0.0001, compared with the model group.

The activation of these immune cells is pivotal for mounting an effective antiviral defense within the intestinal tract. In the piglets infected with PEDV, fluorescence signals for CD4^+^ and CD8^+^ T cells (red fluorescence) in the intestinal tract are significantly diminished. Treatment leads to a notable enhancement of these signals (Figure 8A–D). Conversely, CD127 expression on Tregs is significantly elevated in piglets after PEDV infection, with levels markedly reduced following treatment (Figure 8E,F). These findings indicate that PEDV infection results in a substantial reduction of CD4^+^ and CD8^+^ T cells and impaired Treg functions, suggesting an immune tolerant or suppressive state in the intestinal immune environment. In contrast, CMCTs effectively modulate immune cell populations in the intestinal tract, enhancing immune barrier functions, promoting viral clearance, and reducing inflammation.

### 3.10. CMCTs Regulated the Composition of Gut Microbiota

**Figure 9:**
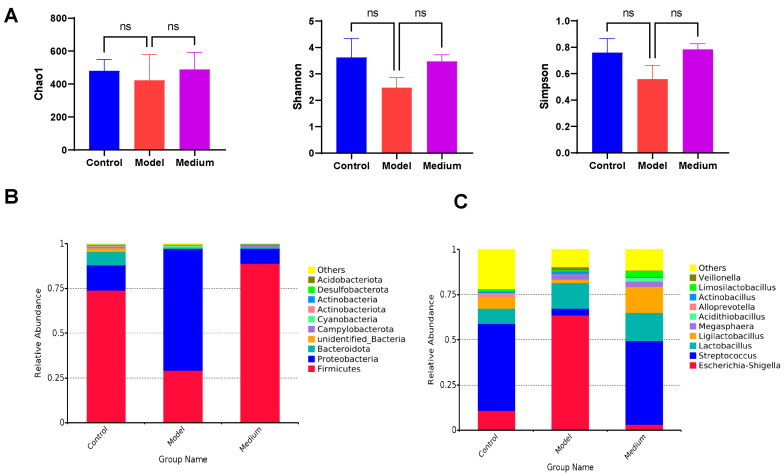
CMCTs regulated the composition of intestinal microbiota. (**A**) Difference in alpha diversity, demonstrated by Chao1, Shannon, and Simpson indexes. Differences in relative microbial abundance between the two groups at the phylum level (**B**) and the genus level (**C**). Statistical significance was determined using one-way ANOVA, followed by Dunnett’s post hoc test. ns, not significant; * *p* < 0.05, ** *p* < 0.01, *** *p* < 0.001, **** *p* < 0.0001, compared with the model group.

Using 16S rRNA gene sequencing, we observed how CMCTs affected the gut microbiota composition of the PEDV-infected pigs. PEDV infection alters intestinal microbiota composition and diversity through inflammation, oxidative stress, and immune dysregulation, which exacerbates intestinal damage and hinders recovery. Results indicate shifts in microbiota diversity (Figure 9A) and relative species abundance in the jejunal contents of the model group post-infection. Specifically, the abundance of *Firmicutes* decreases, while *Proteobacteria* increases at the phylum level (Figure 9B). At the genus level, the abundance of *Escherichia-Shigella* sharply increases, while the abundance of *Streptococcus* and *Ligilactobacillus* decreases (Figure 9C). After treatment, the microbiota composition in treated piglets significantly differs from the model group and more closely resembles that of healthy piglets.

## 4. Discussion

PEDV infects pigs of all ages, with a 100% mortality rate in infected suckling piglets. Analysis of fecal and intestinal tissue samples from diarrheal pigs identified PEDV as the most prevalent virus in recent years in China, with a prevalence rate of 61.8%. Additionally, PEDV strains circulating in China exhibit higher recombination frequency and faster evolutionary rate than those from other regions [26]. This finding may clarify the limited efficacy of current PED vaccines, highlighting the necessity for the swine industry to investigate effective therapeutic options to safeguard pigs against PEDV infection. In this study, we found that CMCTs alleviated clinical signs and significantly increased the levels of total IgM, IgG, and IgA, which may indicate an overall enhancement of systemic immune status. IgM is typically the first antibody produced during acute infection and is indicative of early immune activation [27], while IgG and IgA are generally associated with long-term systemic and mucosal immunity, respectively [28]. A limitation of this study is that only total immunoglobulin levels were measured, without assessing anti-PEDV antibodies. To more accurately evaluate the immunomodulatory effects of CMCTs, future studies employing PEDV-specific ELISAs or virus neutralization assays are warranted to determine whether CMCTs elicit virus-specific humoral immune responses.

PEDV infection in neonatal pigs induces severe atrophic enteritis, primarily affecting the jejunum and ileum, and triggers a significant release of inflammatory factors [1], oxidative stress, and disruption of intestinal barrier functions. While moderate inflammation is essential for clearing infections, excessive activation of inflammatory cells and the subsequent release of numerous inflammatory mediators can impede tissue repair [29]. Research findings indicate that CMCTs effectively reduce histopathological lesions in the jejunum and ileum, decrease the expression of pro-inflammatory factors, and increase the expression of anti-inflammatory factors, thereby mitigating intestinal damage caused by excessive inflammation. Additionally, CMCTs enhance intestinal antioxidant capacity and elevate the expression of the intestinal mucosal repair factor IL-22, promoting intestinal damage repair.

The induction of IFN-α and IFN-γ plays a pivotal role in the host’s innate immune defense against viral pathogens. Previous studies have demonstrated that PEDV infection suppresses type I and II IFN induction in vivo [30,31]. Consistent with these findings, our results show that CMCTs significantly enhance IFN-α and IFN-γ expression by intestinal immune cells, promoting viral clearance by intestinal epithelial cells.

The intestinal barrier can be divided into a mechanical barrier, biological barrier, chemical barrier, and immune barrier [32]. claudin-1, occludin, and ZO-1 are essential components of the structural and functional units of tight junctions (TJs) [33], and play an important role in intestinal mechanical barrier function [34]. Our results showed that CMCTs enhance the expression of claudin-1 and ZO-1 in intestinal tissue. Additionally, CMCTs upregulate MUC-2 and sIgA levels and modulate immune cell populations, strengthening both the intestinal chemical barrier and mucosal immunity, thereby enhancing resistance to viral infection and mitigating virus-induced intestinal damage.

The intestinal microbiota is a vital component of the gut’s biological barrier, playing a key role in maintaining homeostasis and defending against pathogenic infections [35]. In this study, PEDV infection significantly disrupted the ecological balance of the gut microbiota in piglets, as evidenced by decreased microbial diversity, a reduction in the relative abundance of *Firmicutes*, and an increase in *Proteobacteria*. These alterations are consistent with previous reports describing gut dysbiosis following PEDV infection [36,37]. Growing evidence suggests that the intestinal microbiota and its metabolites actively contribute to resistance against PEDV infection through multiple mechanisms, including modulation of mucosal immunity [38], and reinforcement of the intestinal barrier [39,40,41]. Our results further revealed a marked increase in pathogenic genera such as *Escherichia-Shigella*, alongside a reduction in beneficial genera including *Streptococcus* and *Ligilactobacillus*. The overgrowth of *Escherichia-Shigella* has been linked to intestinal inflammation and barrier dysfunction [42], while *Ligilactobacillus* species are known to support gut health and immune modulation [43], and have also been implicated in the maintenance of overall animal health [44]. These findings suggest that maintaining a balanced microbial community may be critical for mitigating PEDV-induced intestinal damage and promoting recovery. Treatment with CMCTs significantly modulated the gut microbiota composition, shifting it toward a profile more closely resembling that of healthy piglets. This modulation is likely to promote gastrointestinal health by supporting a balanced and stable microbial ecosystem. However, the specific mechanisms by which CMCTs regulate individual bacterial genera remain unclear and require further investigation.

Based on the above discussion, CMCTs demonstrate a pronounced protective effect against PEDV-induced lesions. The mechanisms primarily involve anti-inflammatory, antioxidant, and intestinal barrier regulation functions, all contributing to enhancing the endogenous antiviral capacity, promoting viral clearance, and assisting the organism in improving recovery capabilities, thus exerting therapeutic effects against PEDV. Nevertheless, despite these promising findings, certain limitations of the study should be acknowledged. The relatively small sample size, limited observation period, and specific aspects of the experimental design may restrict the generalizability and robustness of the conclusions. Consequently, further research involving larger cohorts and extended clinical evaluations is necessary to comprehensively validate the protective efficacy of CMCTs and to support their translation into practical applications. A limitation of the present study is the absence of direct viral load measurements, which restricts definitive demonstration of CMCTs’ antiviral effects in this model. Although previous research has documented the antiviral activity of CMCTs, and our results indicate improvements in clinical and immunological parameters, future investigations incorporating quantitative viral assays, such as RT-qPCR, are warranted to strengthen mechanistic insights. Moreover, the precise antiviral mechanisms of CMCTs remain to be elucidated. Future studies focusing on the molecular and immunological pathways involved will be essential to provide a stronger theoretical foundation for the development and clinical use of CMCT-based interventions.

## Figures and Tables

**Table 1:** Statistics of clinical signs in each group after PEDV infection.

Groups	Signs	Time After Infection (Days)						
		1	2	3	4	5	6	7
Control	diarrhea	0/5	0/5	0/5	0/5	0/5	0/5	0/5
	Dehydration	0/5	0/5	0/5	0/5	0/5	0/5	0/5
	Vomiting	0/5	0/5	0/5	0/5	0/5	0/5	0/5
Model	diarrhea	0/5	3/5	4/5	5/5	4/4	3/3	3/3
	dehydration	0/5	2/5	4/5	5/5	4/4	3/3	3/3
	vomiting	0/5	3/5	4/5	5/5	4/4	3/3	3/3
Low	diarrhea	0/5	3/5	4/5	5/5	5/5	5/5	4/5
	dehydration	0/5	2/5	4/5	4/5	4/5	4/5	4/5
	vomiting	0/5	3/5	3/5	5/5	4/5	4/5	4/5
Medium	diarrhea	0/5	3/5	3/5	3/5	1/5	1/5	0/5
	dehydration	0/5	1/5	2/5	1/5	1/5	1/5	0/5
	vomiting	0/5	0/5	0/5	0/5	0/5	0/5	0/5
High	diarrhea	0/5	3/5	3/5	2/5	1/5	1/5	0/5
	dehydration	0/5	1/5	1/5	1/5	1/5	0/5	0/5
	vomiting	0/5	0/5	0/5	0/5	0/5	0/5	0/5

## Data Availability

The datasets generated and analyzed during the current study are available in the China National Centre for Bioinformation Repository (GSA: CRA018805) and are publicly accessible at https://ngdc.cncb.ac.cn/gsub/submit/gsa/subCRA030312/finishedOverview (accessed on 9 April 2025).
